# Supplementary material for: Enzymatic Hydrolysis of Resorcylic Acid Lactones by an Aeromicrobium sp
Source: Toxins (Basel). 2024 Sep 19;16(9):404. doi: 10.3390/toxins16090404 (PMC11435890; doi:10.3390/toxins16090404)
Supplement: Supplementary file 1 [file toxins-16-00404-s001.zip › toxins-3140492-supplementary.pdf]

Supplementary Materials:Enzymatic hydrolysis of resorcylic acid lactones by an *Aeromicrobium* sp.

Shawn J. Hoogstra, Kyle N. Hendricks, David R. McMullin, Justin B. Renaud, Juhi Bora, Mark W. Sumarah, and Christopher P. Garnham

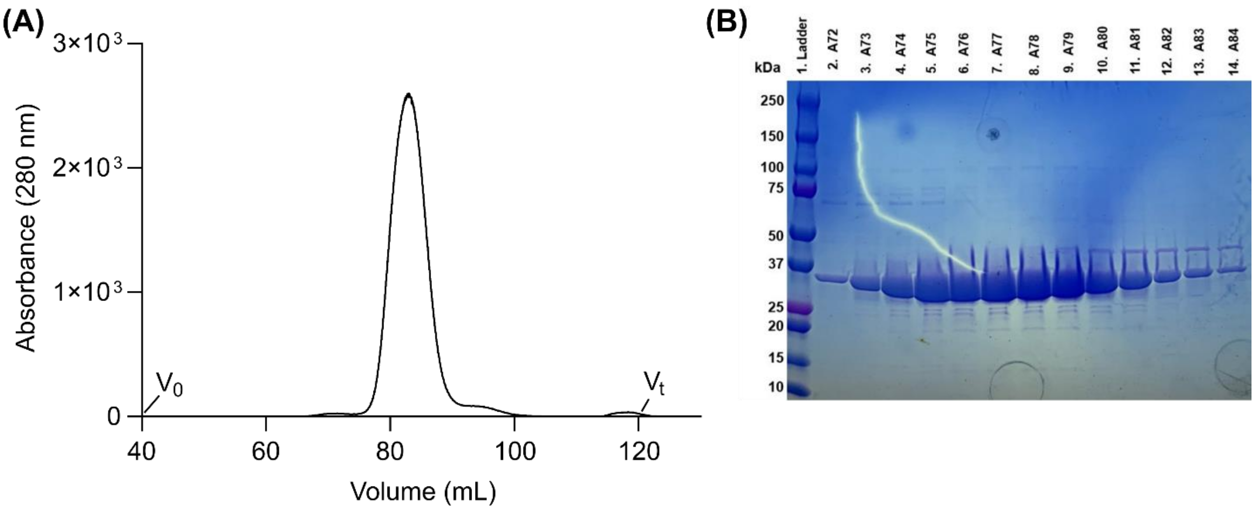

Figure S1. (A) Gel permeation chromatogram of recombinant RALH. Black line represents absorbance at 280 nm. V_o and V_t represent void volume and total volume of the column respectively. (B) SDS–PAGE analysis of RALH fractions spanning the entire elution peak in (A). Ladder = protein markers. Numbers down the left side of the gel represent molecular weight (kDa) of the protein markers.

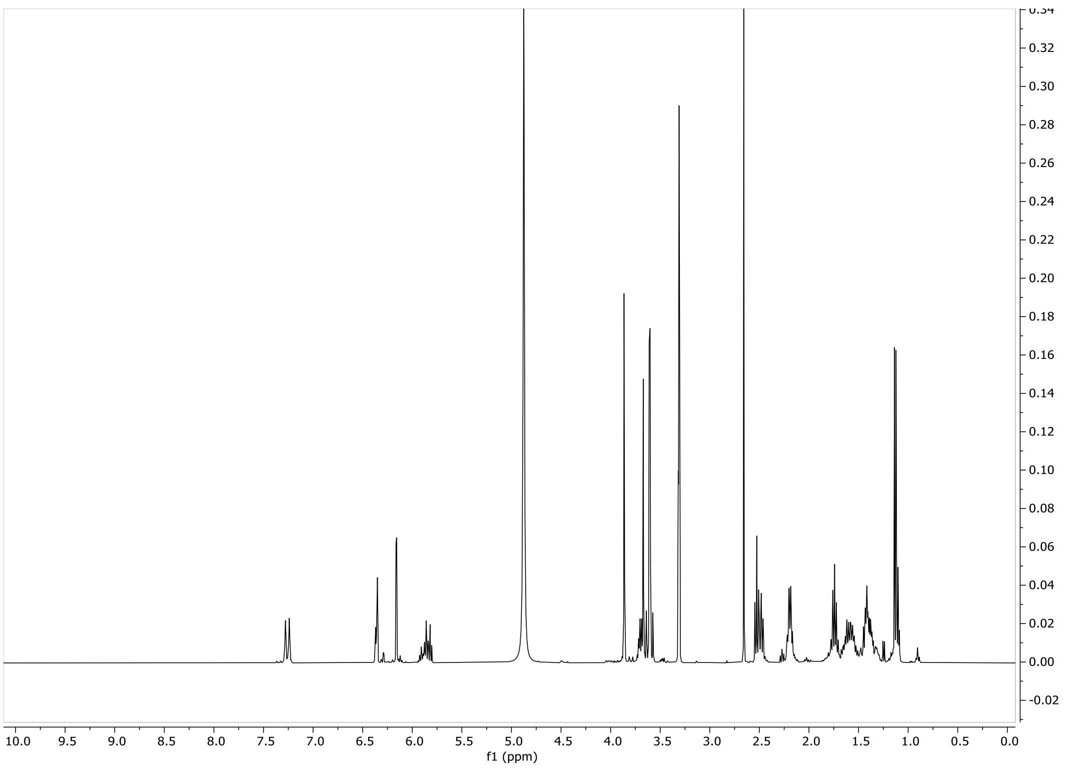

Figure S2. ^{1}H (400 MHz) NMR spectrum for HZEN in CD_3OD

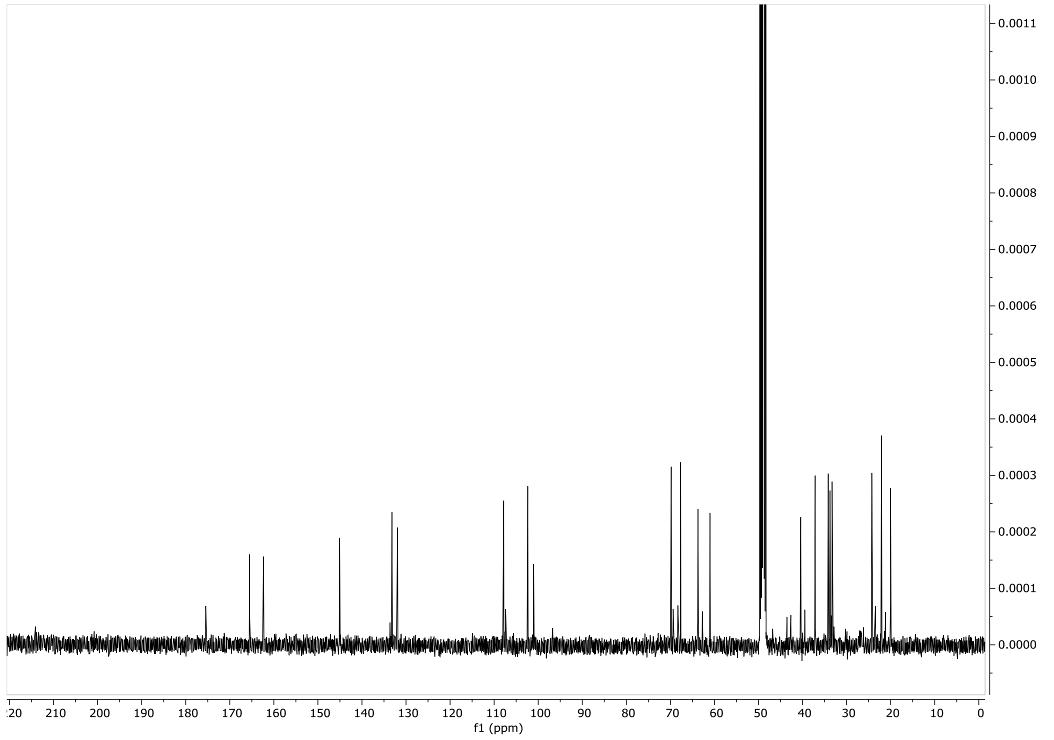

Figure S3. ^{13}C (100 MHz) NMR spectrum for HZEN in CD_3OD

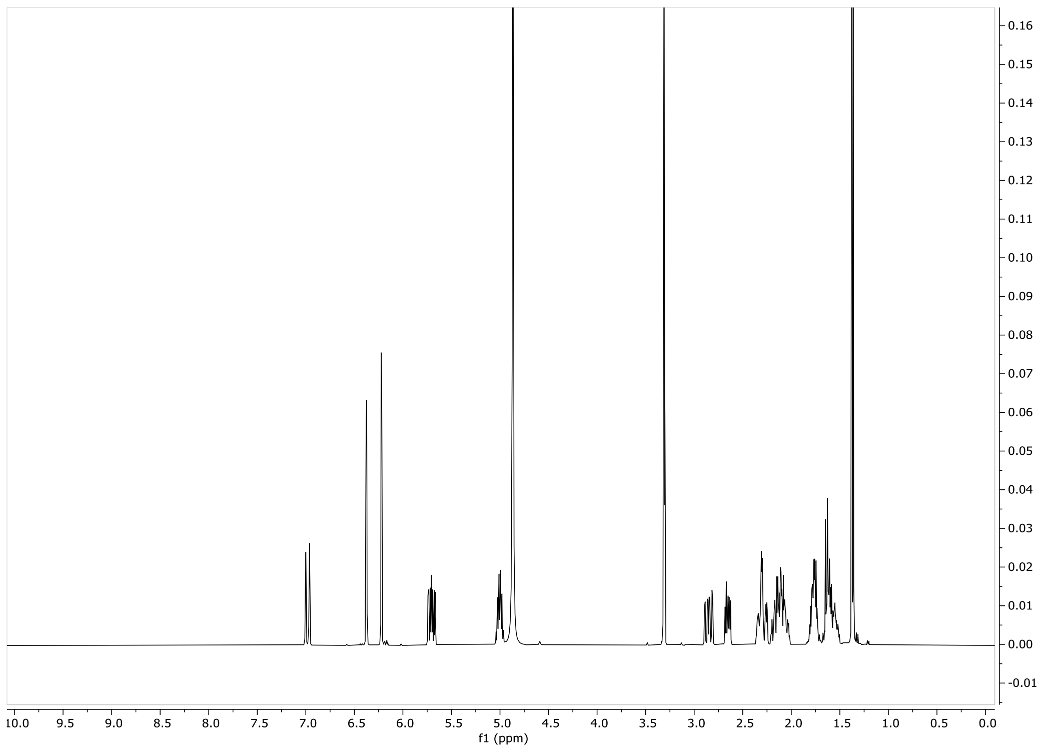

Figure S4. ^{1}H (400 MHz) NMR spectrum for ZEN in CD_3OD

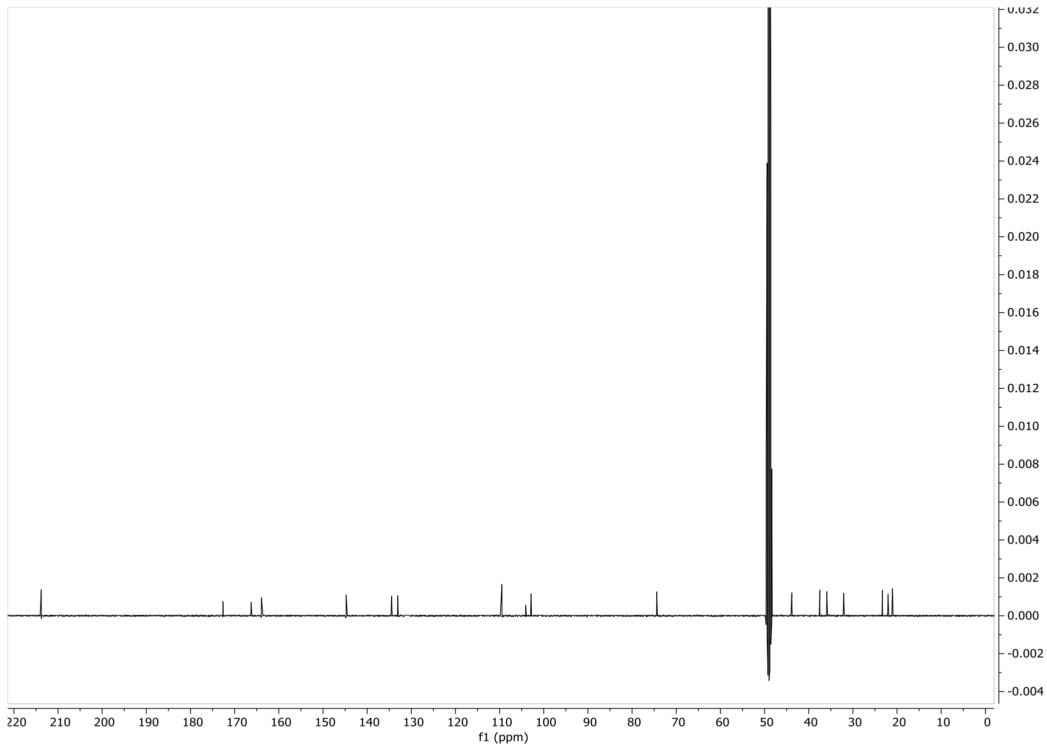

Figure S5. ^{13}C (100 MHz) NMR spectrum for ZEN in CD_3OD

(A)

```
Aero. Sp. HA        MNDRSPTRHGAPDDVFVAHGFEEKLVNLGEIDMNYAEAGSPTKPALLLLPSQSESWWGYE     60
Aero. Sp. LRDC-1    MNDKSPTRRGAPDEVFVAHGFDEKLVNLGEIDMNYAEAGTPSKPALLLLPSQSESWWGYE     60
                    ***:****:****:*******:*****************:*:******************

Aero. Sp. HA        EVMHLLTDDFHVFAVDMRGQGRSTWTPGRYSLDNFGNDLVRFIDQVIGRPVIVAGNSSGG     120
Aero. Sp. LRDC-1    EVMRLLADDFHVFAVDMRGQGRSTWTPGRYSLDNFGNDLVRFIDQVIGRPVIVAGNSSGG     120
                    ***:**:*****************************************************

Aero. Sp. HA        LIAAWLAAYSLPGQIRAAFAEDAPFFASELTPKVGHTIRQAAGHIFVNWRDFLGDQWCVG     180
Aero. Sp. LRDC-1    LIAAWLAAYSLPGQIRAAFAEDAPFFASELTPKVGHTIRQAAGHIFVNWRDFLGDQWCVG     180
                    ************************************************************

Aero. Sp. HA        DYEAYLKAMRNSEIPMLRQVPLPDEAPQNLKEYDAEWARAFYDGTVAQTCPHHTMLAQVK     240
Aero. Sp. LRDC-1    DYSAYLGAMRNSEIPMLRQVPLPDEAPQNLKEYDAEWARAFYDGTVAQTCPHHTMLAQVK     240
                    **.*** *****************************************************

Aero. Sp. HA        APVLVTHHFRLIDPTTAGLMGAMSDLQAEKAMELMREAGVKVDYVDAPDAPHIMHALEPE     300
Aero. Sp. LRDC-1    APVLVTHHFRLIDPTTAGLMGAMSDLQAEKAMELMREAGSKVDYVDLPDAPHIMHALEPE     300
                    *************************************** ****** *************

Aero. Sp. HA        RYVGILRDWVSTLPQA  316
Aero. Sp. LRDC-1    RYVRILRDWVSTLPQA  316
                    *** ************
```

(B)

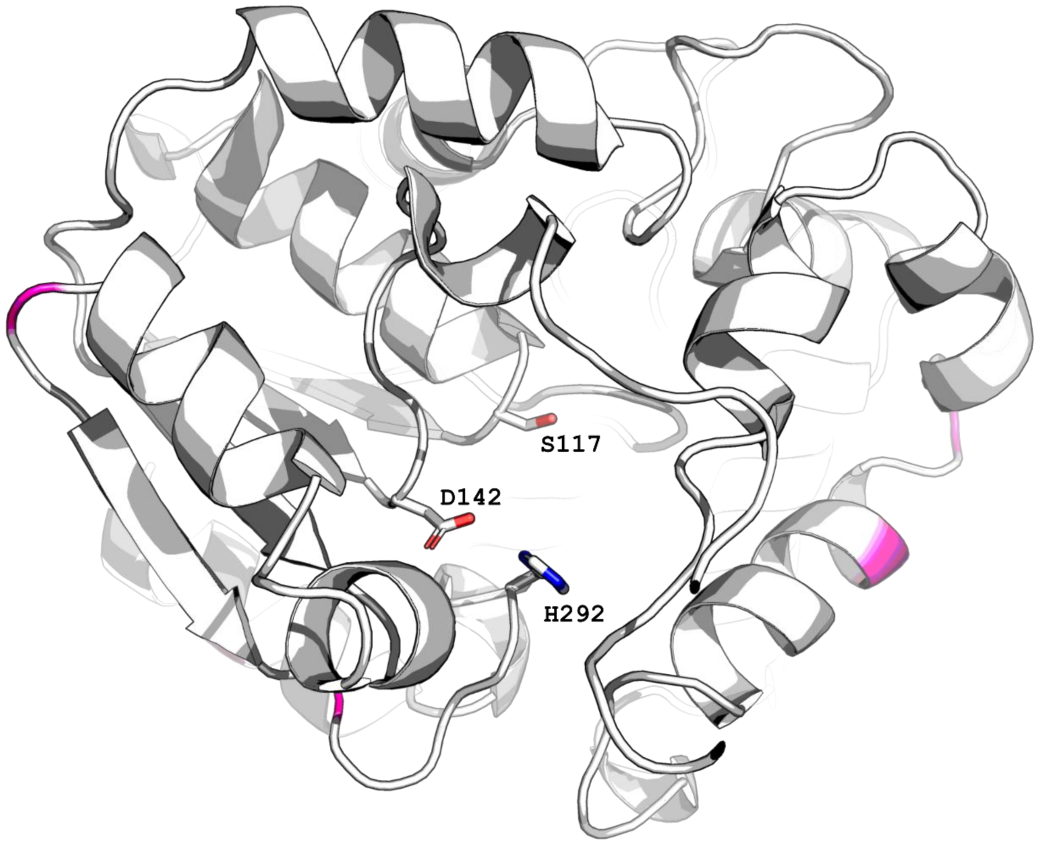

Figure S6. (A) Amino acid alignment between RALH hydrolase identified in this study (LRDC-1) vs. *Aeromicrobium* sp. HA hydrolase identified in [33]. Catalytic triad residues are colored red. * indicates an identical amino acid, while : indicates a strongly conserved substitution, and . represents a weakly conserved substitution. (B**)** Tertiary structure of RALH as predicted by Phyre2 [34], with 100% confidence in the overall architecture of the model. Residues that constitute the catalytic triad of the enzyme are labelled and shown in side-chain stick format. Residues colored purple are substitutions between the two hydrolases and are located at the periphery of the enzyme away the active site.

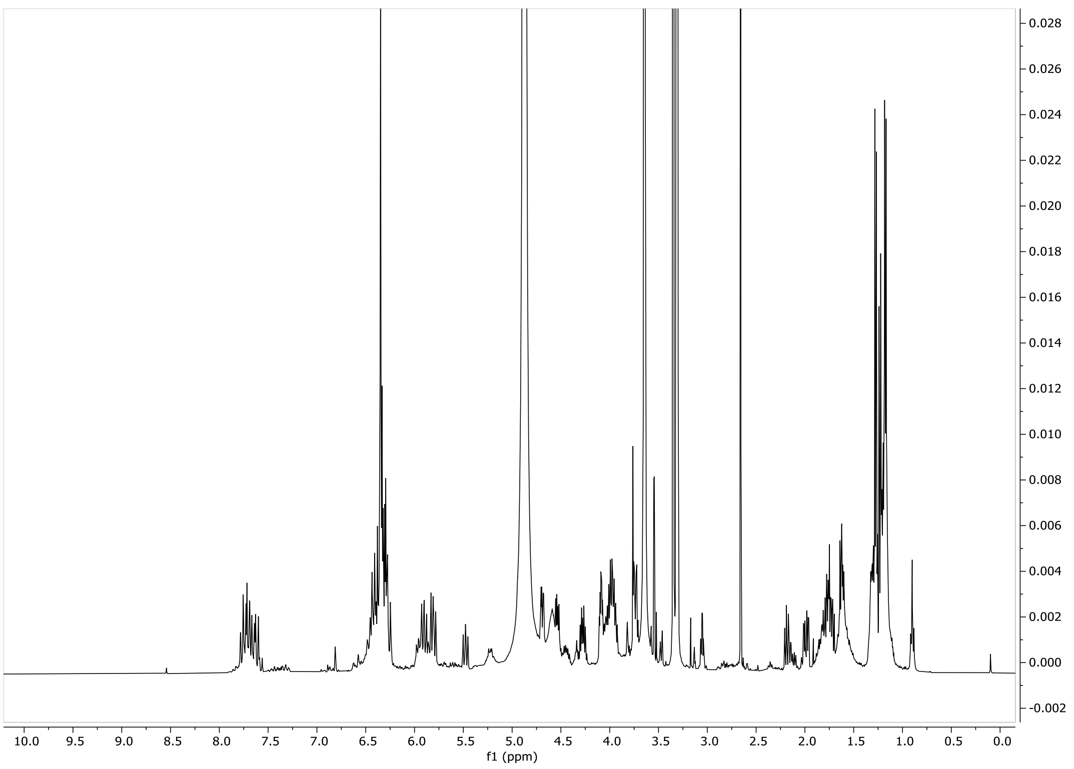

Figure S7. ^{1}H (400 MHz) NMR spectrum for HRAD in CD_3OD

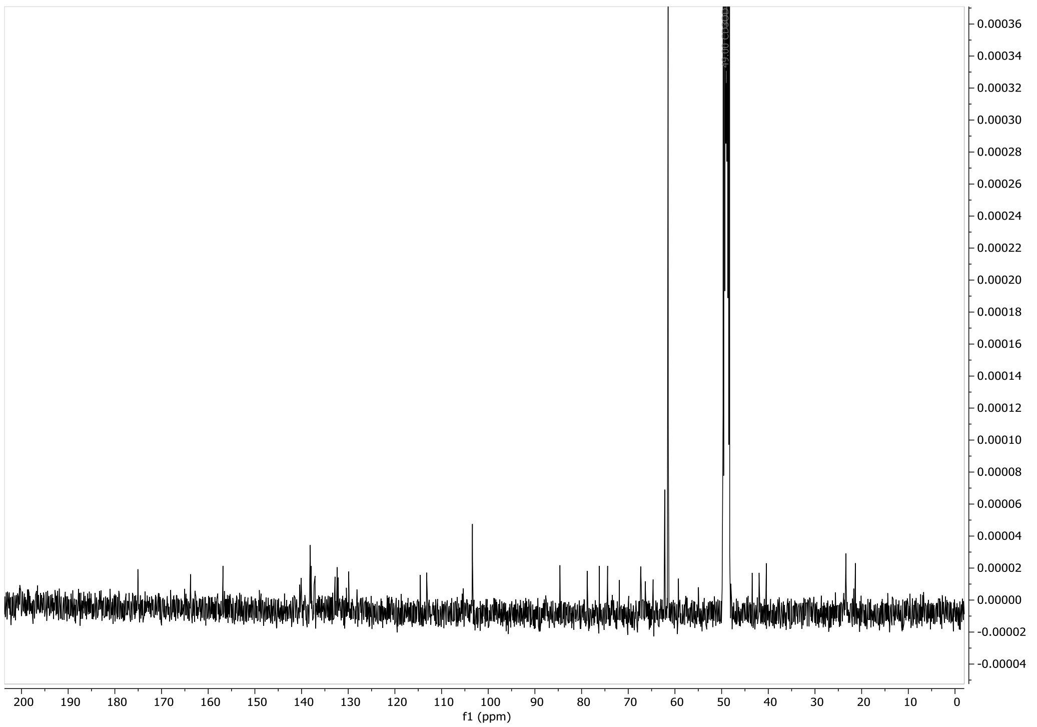

Figure S8. ^{13}C (100 MHz) NMR spectrum for HRAD in CD_3OD

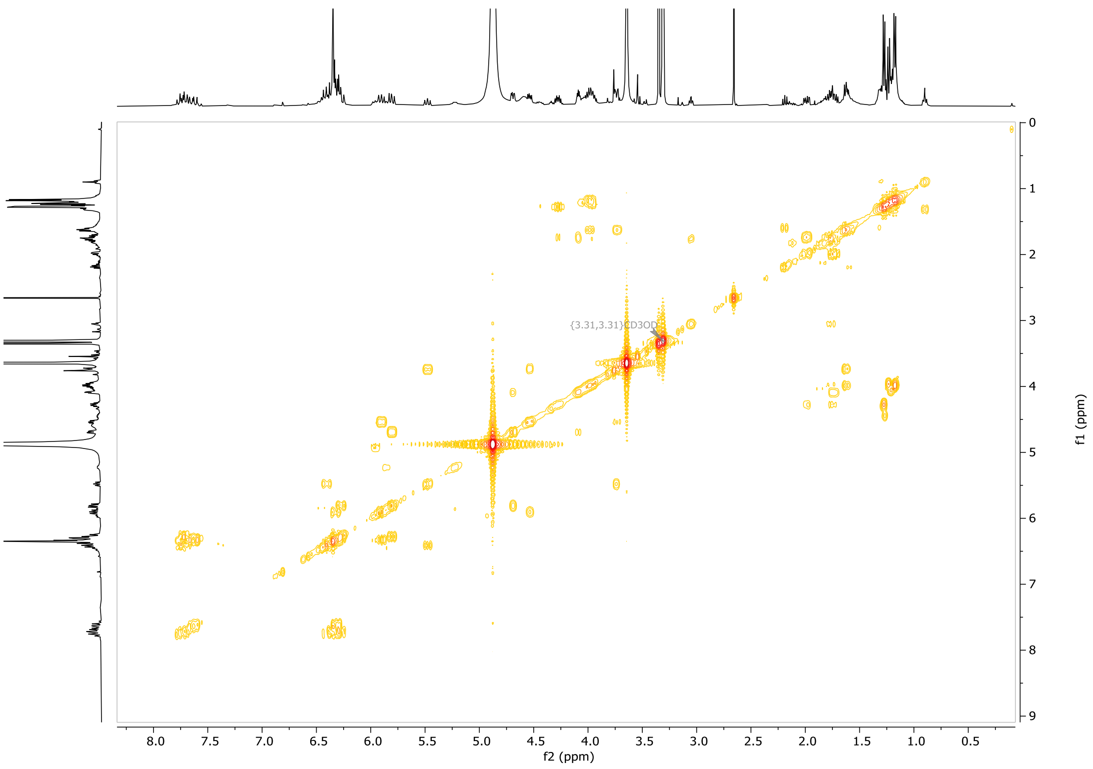

Figure S9. COSY NMR spectrum for HRAD in CD_3OD

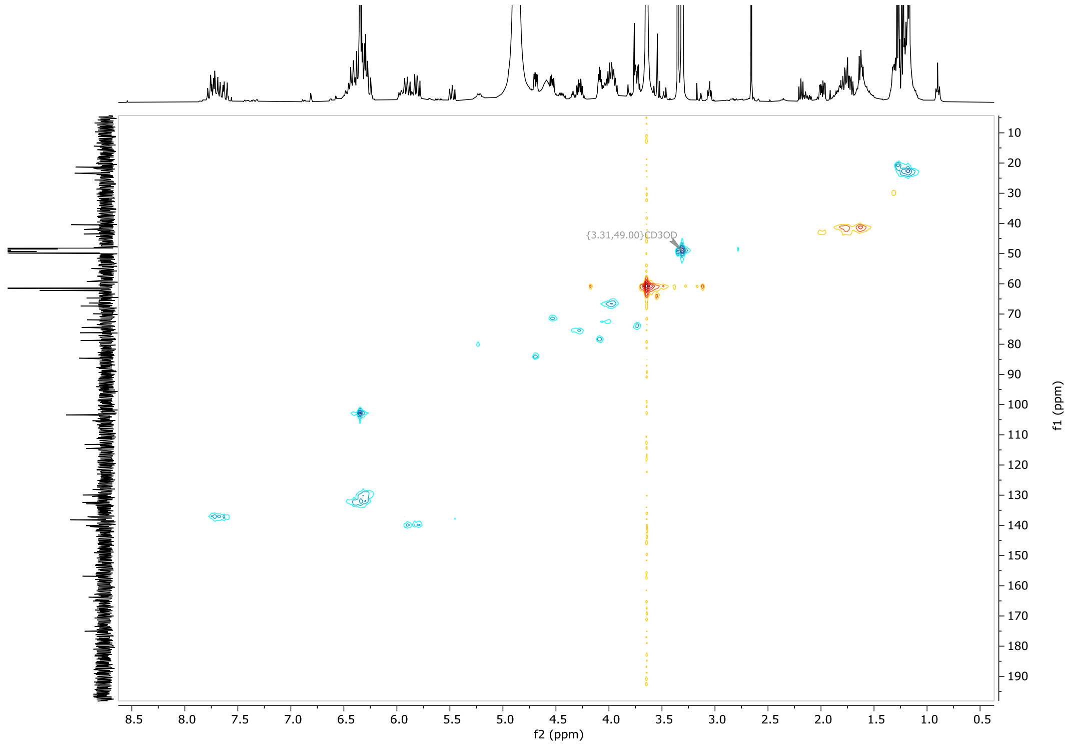

Figure S10. HSQC NMR spectrum for HRAD in CD_3OD

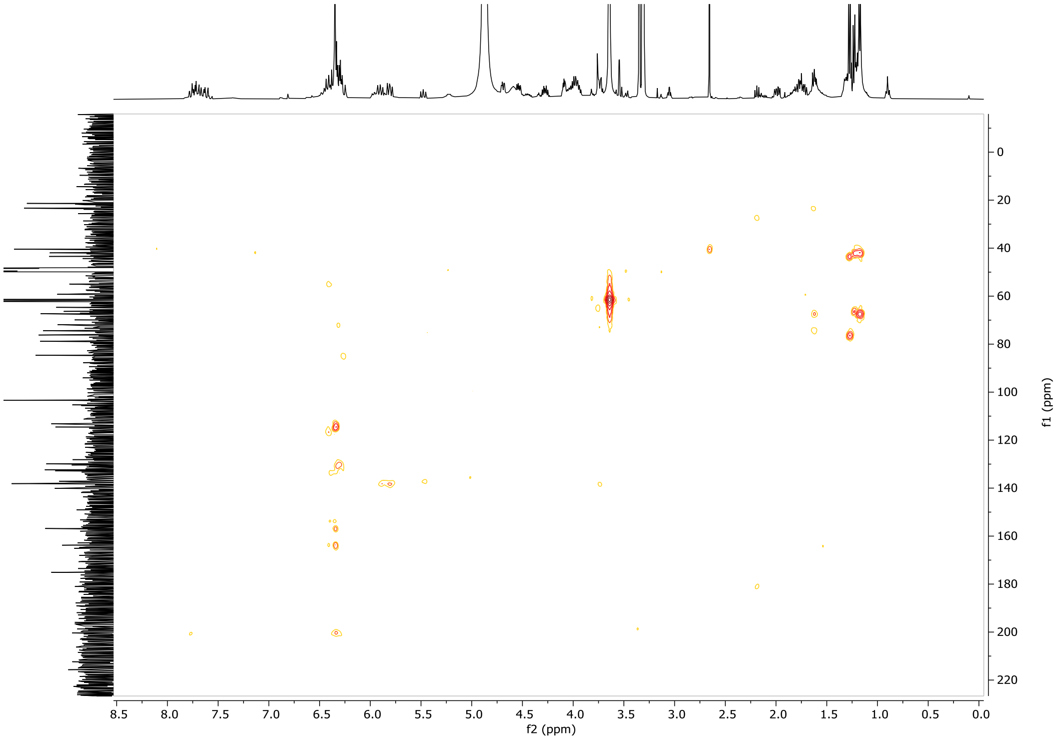

Figure S11. HMBC NMR spectrum for HRAD in CD_3OD

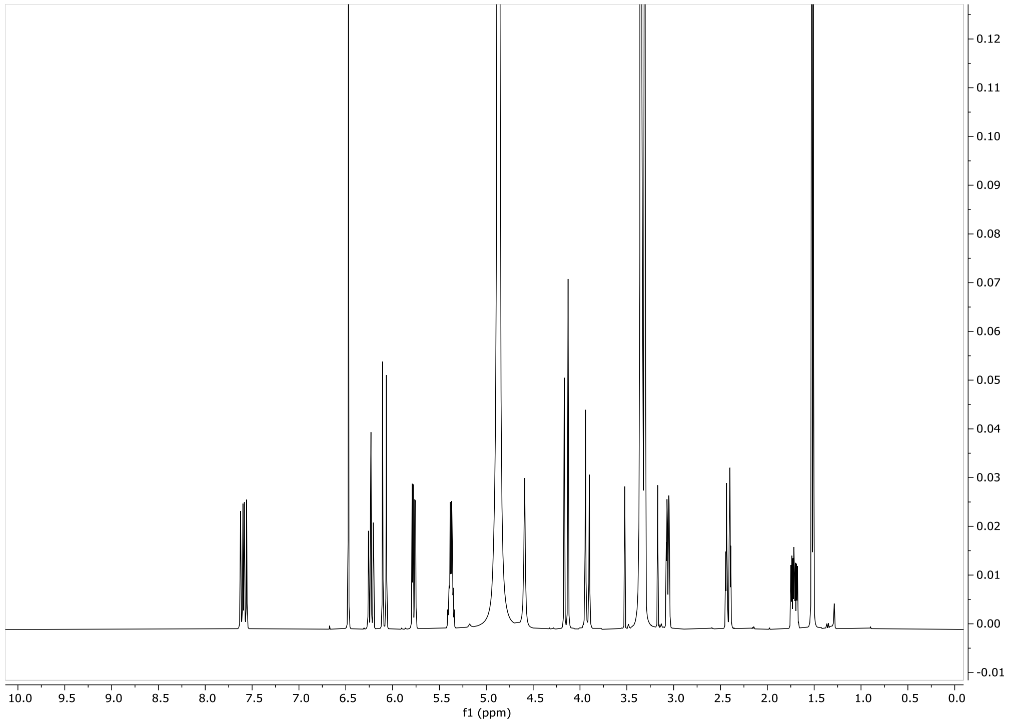

Figure S12. ^1H (400 MHz) NMR spectrum for RAD in CD_3OD

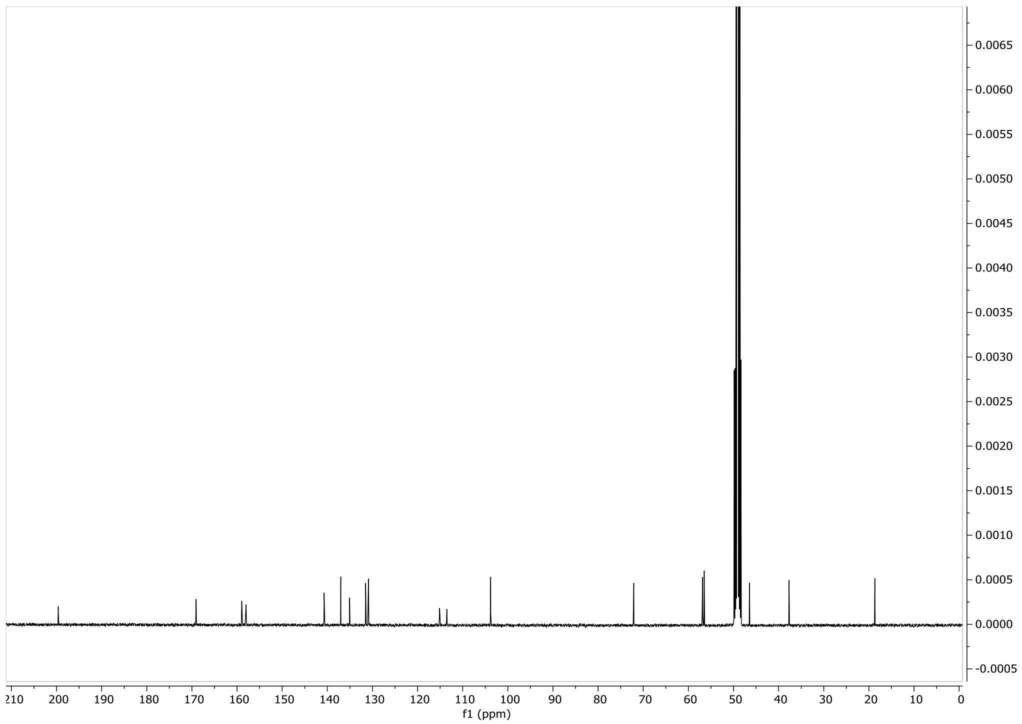

Figure S13. ^{13}C (100 MHz) NMR spectrum for RAD in CD_3OD

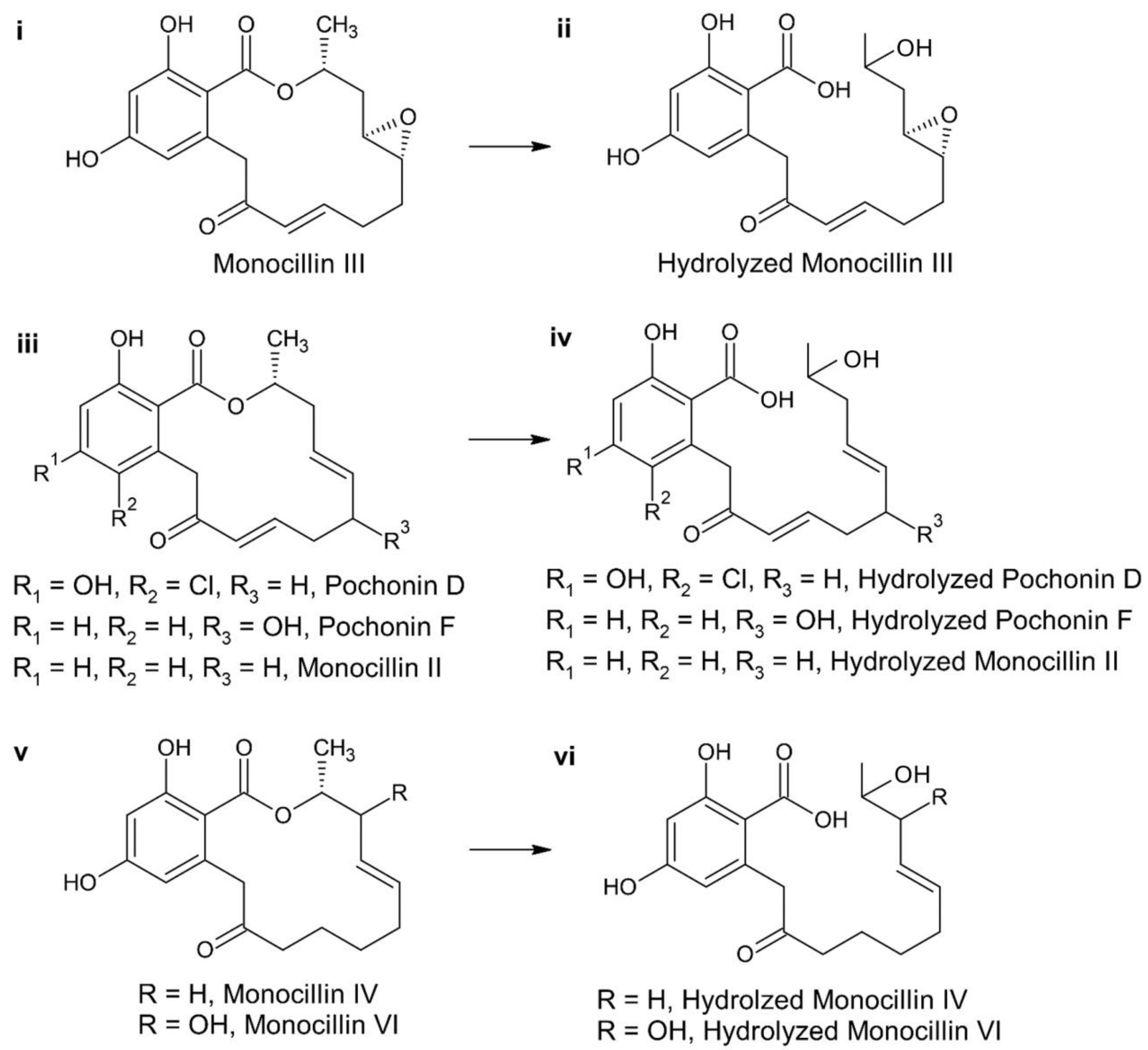

Figure S14. Chemical structures of additional resorcylic acid lactones produced by *Ilyonectria mors-panacis* and hydrolyzed by RALH. **i,** monocillin III; **ii**, hydrolyzed monocillin III; **iii**, pochonin D, pochonin F and monocillin II; **iv**, hydrolyzed pochonin D, pochonin F and monocillin II; **v**, monocillin iv, monocillin vi; **vi**, hydrolyzed monocillin iv and monocillin vi.

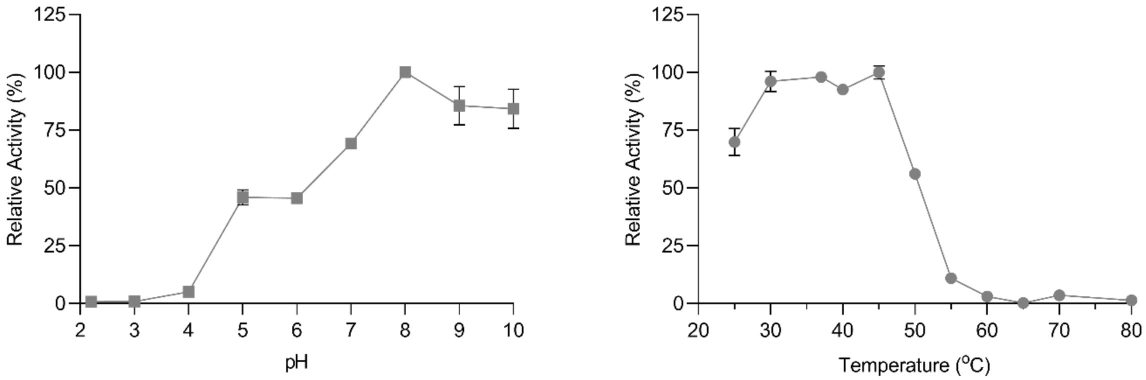

Figure S15. Effect of pH (left) and temperature (right) on RALH ZEN hydrolysis activity as determined via LC-MS analysis. Error bars represent standard deviation (n=3).

Table S1. LC-MS/MS proteomics data of fractions i-vi following Q-Sepharose enrichment of ZEN hydrolysis activity. i-vi = fractions assayed for ZEN hydrolysis activity following Q-Sepharose enrichment (see Figure 3B). LFQ = label free quantification. PCC=Pearson correlation coefficient. The top ranked enzyme candidate, menH_2 (RALH), is colored red.

	Normalized LFQ Intensity						
**Protein IDs**	**i**	**ii**	**iii**	**iv**	**v**	**vi**	**PCC**
sp|menH_2	0.002	0.449	1.000	0.586	0.162	0.071	0.992
sp|valS	0.005	0.523	1.000	0.434	0.068	0.014	0.990
sp|gpmA	0.003	0.434	1.000	0.559	0.098	0.057	0.989
sp|menB	0.023	0.683	1.000	0.530	0.116	0.047	0.986
sp|dapA	0.016	0.546	1.000	0.627	0.067	0.076	0.986
sp|rpsA	0.093	0.702	1.000	0.479	0.240	0.033	0.979
sp|nadE_1	0.000	0.377	1.000	0.495	0.031	0.057	0.977
sp|nuoF	0.094	0.640	1.000	0.603	0.427	0.067	0.977
sp|HBAOAJCG_00853	0.142	0.804	1.000	0.689	0.382	0.171	0.976
sp|HBAOAJCG_02340	0.000	0.633	1.000	0.426	0.000	0.000	0.975
sp|leuS	0.014	0.600	1.000	0.365	0.048	0.005	0.974
sp|apaH	0.124	0.614	1.000	0.794	0.232	0.084	0.973
sp|purE	0.019	0.331	1.000	0.397	0.128	0.011	0.972
sp|aceE	0.113	0.783	1.000	0.547	0.217	0.078	0.971
sp|polA	0.008	0.663	1.000	0.811	0.247	0.045	0.970
sp|dapE_1	0.000	0.321	1.000	0.553	0.047	0.019	0.970
sp|tdh_1	0.039	0.521	1.000	0.336	0.046	0.056	0.967
sp|dop	0.000	0.513	1.000	0.699	0.000	0.081	0.965
sp|guaA	0.095	0.755	1.000	0.442	0.176	0.024	0.962
sp|HBAOAJCG_02438	0.000	0.412	1.000	0.702	0.000	0.000	0.962
sp|glnE	0.005	0.295	1.000	0.583	0.083	0.049	0.960

sp|HBAOAJCG_00727	0.007	0.345	1.000	0.405	0.132	0.142	0.960
sp|gap1	0.157	0.812	1.000	0.533	0.208	0.109	0.959
sp|nuoD	0.012	0.263	1.000	0.393	0.069	0.000	0.958
sp|ribBA	0.000	0.316	1.000	0.641	0.074	0.056	0.957
sp|aidB	0.073	0.378	1.000	0.341	0.000	0.000	0.956
sp|HBAOAJCG_00382	0.000	0.542	1.000	0.299	0.000	0.072	0.955
sp|ephD	0.000	0.276	1.000	0.568	0.000	0.000	0.955
sp|narG	0.094	0.701	1.000	0.373	0.091	0.000	0.954
sp|bcs1	0.002	0.409	1.000	0.777	0.101	0.058	0.952
sp|dnaE1	0.021	0.279	1.000	0.654	0.077	0.017	0.946
sp|HBAOAJCG_01706	0.000	0.243	1.000	0.334	0.158	0.000	0.944
sp|HBAOAJCG_01619	0.000	0.298	1.000	0.649	0.297	0.024	0.943
sp|HBAOAJCG_01083	0.106	0.586	1.000	0.470	0.504	0.178	0.942
sp|HBAOAJCG_02805	0.000	0.772	1.000	0.358	0.000	0.006	0.940
sp|HBAOAJCG_03127	0.000	0.310	1.000	0.468	0.158	0.227	0.935
sp|ecm	0.066	0.758	1.000	0.961	0.293	0.098	0.935
sp|HBAOAJCG_01952	0.000	0.295	1.000	0.647	0.314	0.100	0.935
sp|HBAOAJCG_03237	0.139	0.589	1.000	0.858	0.138	0.260	0.933
sp|recB	0.084	0.232	1.000	0.477	0.000	0.000	0.932
sp|HBAOAJCG_01591	0.024	0.310	1.000	0.756	0.322	0.077	0.922
sp|moaC2	0.085	0.928	1.000	0.639	0.000	0.000	0.920
sp|nuoB	0.000	0.304	1.000	0.153	0.000	0.000	0.918
sp|COQ5_2	0.049	0.185	1.000	0.595	0.000	0.000	0.917
sp|clpB	0.005	0.479	1.000	0.129	0.048	0.017	0.917
sp|iscS_1	0.176	0.511	1.000	0.914	0.124	0.038	0.915
sp|HBAOAJCG_01190	0.000	0.259	1.000	0.155	0.000	0.000	0.911
sp|aldA	0.056	0.724	1.000	0.233	0.018	0.041	0.910
sp|leuA	0.013	0.962	1.000	0.423	0.024	0.005	0.905
sp|caiD_1	0.000	0.183	1.000	0.727	0.000	0.000	0.904
sp|HBAOAJCG_01455	0.277	0.843	1.000	0.531	0.064	0.186	0.900
sp|grpE	0.108	0.992	1.000	0.471	0.129	0.059	0.899
sp|nuoG	0.094	0.433	1.000	0.912	0.403	0.252	0.895
sp|dltA	0.000	0.168	1.000	0.765	0.000	0.000	0.891
sp|tyrS	0.047	0.545	1.000	0.729	0.652	0.119	0.888
sp|xseB	0.321	0.689	1.000	0.429	0.000	0.072	0.885
sp|pepE_1	0.070	0.757	1.000	0.641	0.375	0.568	0.876
sp|fabG_1	0.000	0.066	1.000	0.627	0.145	0.000	0.875
sp|gatA	0.057	0.536	1.000	0.763	0.335	0.537	0.868
sp|nadD	0.000	0.481	1.000	0.025	0.000	0.070	0.862
sp|yhfQ	0.000	0.345	1.000	0.776	0.000	0.348	0.861
sp|hpd	0.002	0.199	1.000	0.817	0.342	0.133	0.856
sp|HBAOAJCG_02260	0.000	0.874	1.000	0.129	0.000	0.000	0.856
sp|tkt_2	0.000	0.162	1.000	0.836	0.273	0.084	0.854

sp|deoA	0.003	0.220	1.000	0.921	0.328	0.120	0.843
sp|punA	0.000	0.257	1.000	0.964	0.327	0.161	0.840
sp|HBAOAJCG_00360	0.242	0.875	1.000	0.230	0.190	0.000	0.838
sp|HBAOAJCG_01538	0.013	0.100	1.000	0.860	0.209	0.015	0.836
sp|topA	0.093	0.754	1.000	0.555	0.047	0.536	0.830
sp|trkA_1	0.129	0.603	1.000	0.300	0.000	0.406	0.825
sp|carB_1	0.203	0.824	1.000	0.887	0.752	0.633	0.816
sp|atpD	0.111	0.488	1.000	0.834	0.668	0.454	0.815
sp|prmC_3	0.000	0.213	1.000	0.948	0.445	0.098	0.814
sp|bglA_1	0.000	0.157	1.000	0.717	0.000	0.325	0.814
sp|dnaN	0.000	0.105	1.000	0.854	0.366	0.078	0.809
sp|HBAOAJCG_01884	0.031	0.266	1.000	0.933	0.502	0.207	0.809
sp|ppk	0.000	0.034	1.000	0.735	0.263	0.135	0.806
sp|dapE_3	0.206	0.979	1.000	0.392	0.348	0.463	0.804
sp|baiA_2	0.000	0.088	1.000	0.993	0.177	0.048	0.799
sp|HBAOAJCG_01454	0.344	0.821	1.000	0.211	0.000	0.000	0.795
sp|glgE	0.028	0.964	1.000	0.073	0.000	0.152	0.783
sp|pheT	0.486	0.680	1.000	0.336	0.000	0.000	0.782
sp|pheA	0.010	0.939	1.000	0.018	0.243	0.033	0.781
sp|mutB	0.139	0.977	1.000	0.213	0.036	0.286	0.779
sp|ilvG_1	0.017	0.371	1.000	0.870	0.615	0.428	0.777
sp|HBAOAJCG_01753	0.000	0.189	1.000	0.283	0.612	0.000	0.756
sp|nuoC	0.291	0.414	1.000	0.212	0.115	0.366	0.753
sp|HBAOAJCG_02249	0.015	0.345	1.000	0.996	0.758	0.129	0.747
sp|HBAOAJCG_00067	0.407	0.079	1.000	0.690	0.000	0.000	0.726
sp|HBAOAJCG_03270	0.046	0.136	1.000	0.453	0.537	0.331	0.717
sp|ispG	0.000	0.414	1.000	0.584	0.000	0.700	0.696
sp|glmM	0.061	0.222	1.000	0.774	0.553	0.529	0.688
sp|glnA2	0.002	0.275	1.000	0.952	0.513	0.591	0.677
sp|aroC	0.554	0.969	1.000	0.079	0.070	0.009	0.624
sp|mch	0.057	0.239	1.000	0.624	0.661	0.620	0.595
sp|comM	0.000	0.411	1.000	0.411	0.302	0.960	0.471
sp|HBAOAJCG_00749	0.981	0.566	1.000	0.505	0.148	0.162	0.444
sp|typA	0.000	0.476	1.000	0.238	0.261	1.000	0.412

Table S2. ^1H and ^{13}C NMR data for zearalenone and hydrolyzed-zearalenone produced by RALH in CD_3OD

	Zearalenone		Hydrolyzed-zearalenone	
**Position**	**δ_C, type**	**δ_H (J in Hz)**	**δ_C, type**	**δ_H (J in Hz)**
1	22.0, CH_3	1.37, d (6.1)	22.1, CH_3	1.13, d (6.3)
2	74.4, CH	5.00, m	67.7, CH	3.69, m
3	36.0, CH_2	1.61, m	39.5, CH_2	1.39, m
4	21.0, CH2	2.08, m	21.2, CH_2	1.78, m
		1.55, m		1.59, m
5	37.5, CH2	2.84 ddd (18.8, 11.9, 2.6)	43.5, CH_2	2.48, m
		2.28, m		
6	213.8, C		214.1, C	
7	43.9, CH2	2.65, m	42.6, CH_2	2.53, t (7.2)
		2.11, m		
8	23.3, CH2	1.76, m	24.3, CH_2	1.74, p (7.2)
9	32.1, CH2	2.30, m	33.7, CH_2	2.19, m
		2.14, m		
10	133.1, CH	5.71, ddd (15.3, 10.2, 4.0)	131.9, CH	5.86, dt (15.5, 7.0)
11	134.5, CH	6.98, dd (15.3, 2.0)	133.2, CH	7.26, d (15.5)
12	144.8, C		145.1, C	
13	109.5, CH	6.38, d (2.5)	107.8, CH	6.35, d (2.4)
14	164.0, C		162.4, C	
15	102.9, CH	6.22, d (2.5)	102.4, CH	6.16, d (2.4)
16	166.3, C		165.5, C	
17	104.1, C		110.0, C	
18	172.7, C		175.5, C	

References

33. Hu, J.; Wang, G.; Hou, M.; Du, S.; Han, J.; Yu, Y.; Gao, H.; He, D.; Shi, J.; Lee, Y.-W.; et al. New Hydrolase from *Aeromicrobium* sp. HA for the Biodegradation of Zearalenone: Identification, Mechanism, and Application. *J. Agric. Food Chem.* **2023**, *71*, 2411–2420. https://doi.org/10.1021/acs.jafc.2c06410.

34. Kelley, L.A.; Mezulis, S.; Yates, C.M.; Wass, M.N.; Sternberg, M.J.E. The Phyre2 web portal for protein modeling, prediction and analysis. *Nat Protoc.* **2015**, 10, 845-858. doi:10.1038/nprot.2015.053.
